# Sex differences in response to a short-term training program intervention in obese adolescents: a plasma metabolomics study

**DOI:** 10.1186/s13293-026-00896-8

**Published:** 2026-04-01

**Authors:** Lin Zhu, Xianyan Xie, Huiguo Wang, Zekai Chen

**Affiliations:** 1 School of Sport and Health, Guangzhou Sport University, Guangzhou, 510500 China; 2 Innovative Research Center for Sports Science in the Guangdong-Hong Kong-Macao Greater Bay Area, Guangzhou Sport University, Guangzhou, 510500 China; 3 Research Center for Innovative Development of Sports and Healthcare Integration, Guangzhou Sport University, Guangzhou, 510500 China

**Keywords:** Sex differences, Short-term training program intervention, Obese adolescents, Metabolomics

## Abstract

**Background:**

This study aims to investigate sex differences in the response to short-term Training Program intervention and the underlying metabolomic mechanisms among obese adolescents.

**Methods:**

A total of 98 obese adolescents underwent a 4-week, strictly controlled short-term Training Program intervention. Pre- and post-intervention measurements included body morphometry, body composition, lipid metabolism, and glucose homeostasis indicators. Plasma samples were analyzed using targeted metabolomics. Linear mixed-effects models (LMM) were employed to identify Sex-differential Responsive Metabolites, with volcano plots utilized to visualize metabolic regulation preferences between sexes. Hierarchical clustering analysis identified co-regulated metabolic modules within the Sex-differential Responsive Metabolites for pathway analysis. Partial least squares (PLS) regression models were constructed separately for males and females to identify metabolic biomarkers capable of predicting changes in clinical indicators.

**Results:**

Post-intervention, both males and females showed significant decreases in weight, body mass index (BMI), chest circumference, waist circumference, hip circumference, waist-to-hip ratio, body water, Body fat mass, fat free mass, skeletal muscle mass, body fat percentage, total cholesterol (TC), triglycerides (TG), and low-density lipoprotein cholesterol (LDL-C) (*P* < 0.001). Additionally, HOMA-β levels significantly decreased only in females (*P* = 0.01). After adjusting for developmental maturity and baseline metabolic risk, the improvements in body fat mass and body fat percentage in males were significantly greater than those in females (both *P* < 0.01), whereas no statistical differences were found in weight and BMI improvements (*P* > 0.05). The change in HOMA-β differed significantly between sexes (*P* = 0.031), with females exhibiting a more pronounced downregulation following the intervention. LMM identified 65 Sex-differential Responsive Metabolites. Hierarchical clustering revealed two co-regulated modules: Module 1 showed a strong upward trend in males but a weak response in females, significantly enriched in the linoleic acid metabolism pathway; Module 2 showed a downward trend in both sexes, but the magnitude of downregulation was more pronounced in males, significantly enriched in pathways related to amino acid catabolism and energy metabolism. PLS model analysis indicated that Sex-differential Responsive Metabolites had predictive capacity for male Fasting Blood Glucose (FBG, R^2^Y = 0.2653,Q^2^ = 0.0091), female FBG (R^2^Y = 0.3552,Q^2^ = 0.1321), and female LDL-c (R^2^Y = 0.3895,Q^2^ = 0.1853).

**Conclusions:**

Obese adolescents exhibit significant sexual dimorphism in their response to short-term Training Program. Under the same standardized training program, males achieve greater fat reduction and appear to leverage a synergistic strategy of fatty acid oxidation and protein sparing, whereas females prioritize amino-acid network fine-tuning. Predictive modeling indicates that sex-differential metabolites can predict glucolipid improvements in females. These findings provide a scientific basis for developing sex-differential exercise prescriptions for metabolic health.

**Supplementary Information:**

The online version contains supplementary material available at 10.1186/s13293-026-00896-8.

## Introduction

Adolescent obesity has emerged as a severe global public health issue in the 21 st century [1]. According to the World Health Organization, the number of obese individuals worldwide surged from 11 million in 1975 to 124 million in 2016 [2], and this trend continues to escalate [3]. Adolescent obesity not only significantly increases the risk of developing type 2 diabetes mellitus [4], hypertension [5], and atherosclerosis [6] in adulthood but also exerts long-term negative effects on individual mental health [7] and cognitive development [8]. Therefore, elucidating the pathophysiological basis of obesity and developing precise, effective intervention strategies are of paramount public health importance.

Among numerous intervention strategies, exercise is considered a crucial component of weight management measures for adolescents due to its multiple benefits, such as improving body composition [9], regulating energy metabolism [10], and enhancing insulin sensitivity [11]. However, metabolic responses to exercise stimuli exhibit sexual heterogeneity [11]. For instance, studies have found that males are superior to females in terms of fat reduction [12]. This discrepancy may be related to evolutionary adaptations, where males are more prone to energy turnover during exercise, while females tend towards energy conservation to maintain reproductive potential [13]. Nevertheless, most previous studies have treated sex merely as a statistical covariate for adjustment, precluding the precise identification of molecular differences in exercise adaptation between the sexes.

To bridge this knowledge gap, it is necessary to delve into the molecular regulation networks underlying exercise intervention in both sexes. Metabolomics enables the high-throughput capture of dynamic changes in small-molecule metabolites, revealing exercise-induced metabolic remodeling networks and thus precisely depicting physiological adaptation trajectories for both sexes [14]. Although studies have confirmed that exercise influences lipid, amino acid, and energy metabolism pathways [15], few have conducted systematic sex-stratified analyses specifically within the obese adolescent population, limiting our understanding of the mechanisms driving sex differences in short-term Training Program intervention. Based on this, the present study aims to investigate sex differences in response to identical short-term Training Program interventions and the underlying metabolic molecular mechanisms in obese adolescents through a four-week, fully enclosed, and supervised standardized short-term Training program combined with targeted metabolomics analysis. These findings will provide a scientific basis for the development of sex-difference precision exercise interventions.

## Methods

### Subjects and grouping

Based on inclusion and exclusion criteria, a total of 103 obese adolescents were recruited from a specialized weight-loss camp. Five participants withdrew during the study due to personal reasons. Ultimately, 98 obese adolescents completed the 4-week short-term Training Program intervention and all related measurements. These 98 participants were divided into a Male group and a Female group for subsequent statistical analysis.

The inclusion criteria were: (1) Age between 10 and 17 years; (2) Body mass index (BMI) ≥ the 95th percentile of the WHO 2007 growth reference data for age and sex [16].

The exclusion criteria were: (1) Use of medications affecting glucose or lipid metabolism (e.g., glucocorticoids, antidepressants) within the past 6 months; (2) Clinical diagnosis of metabolic disorders such as type 2 diabetes mellitus, severe cardiovascular disease, thyroid dysfunction, or hepatic/renal insufficiency; (3) History of smoking or alcohol consumption within the past 6 months.

### Study design

The study protocol strictly adhered to the ethical guidelines of the Declaration of Helsinki and was approved by the Ethics Committee of Guangzhou Sport University (Ethics Approval No. 2018LCLL-008). Written informed consent was obtained from all subjects and their legal guardians after they were fully informed of the study’s purpose, potential risks, and benefits.

All subjects completed a 4-week, fully enclosed, and supervised standardized short-term Training Program intervention under the strict supervision of professional strength and conditioning coaches. The protocol was as follows:

Frequency: Twice daily (morning and afternoon).

Intensity: 50%–80% of maximum heart rate (HR_max_).

Time: 120 min per session, consisting of a 30-minute warm-up, 80 min of formal training, and a 10-minute cool-down.

Type: The warm-up included dynamic stretching and low-intensity aerobic exercise; formal training combined moderate-intensity aerobic exercise with short-duration high-intensity exercise, including brisk walking, jogging, sports competitions, and recreational ball games; the cool-down involved static stretching and relaxation activities.

Throughout the training process, the Polar heart rate monitoring system (BoHaoTong, China) was used to monitor and record the heart rate of each subject in real-time, ensuring that the heart rate was maintained at 50%–80% of HR_max_ (HR_max_ =220 − age) to meet the target training intensity.The exercise intensity and volume in this study (50–80% HRmax) significantly exceed the recommendations of general public health guidelines, such as those provided by the World Health Organization (WHO) [17]. This regimen was selected for the following reasons: First, metabolomics research is frequently susceptible to substantial inter-individual biological noise. To capture statistically significant, sex-differential responsive metabolic trajectories within a relatively short 4-week intervention, a sufficient “metabolic perturbation” must be applied. Our protocol was designed as a “metabolic stress test,” aimed at pushing the physiological system toward its metabolic limits through intensive stimuli. This approach amplifies subtle sex differences in substrate utilization and energy compensatory mechanisms. While moderate-intensity continuous training (MICT) is effective for general health, the omics signal intensity it induces might be insufficient to overcome baseline biological noise in the short term. Second, the protocol refers to the clinically established model of Residential Weight Loss Programs (RWL) [18]. In RWL settings (e.g., specialized weight-loss camps for adolescents), moderate-to-high intensity exercise combined with rigorous dietary monitoring has been empirically validated as a safe and highly efficient strategy for improving metabolic health in obese adolescents within a short period. Furthermore, our research group has observed in long-term clinical practice that under standard exercise volumes, sex differences in weight-loss phenotypes are often masked by individual variability. To investigate the molecular mechanisms underlying why males typically exhibit more rapid fat loss than females, it was essential to establish an extreme model with a high signal-to-noise ratio.

To eliminate dietary interference with the experimental outcomes, a strictly standardized and individualized dietary intervention was implemented [19]. This protocol follows a established paradigm within our research group and has been validated in previous studies.Initially, the Resting Metabolic Rate (RMR) of each participant was accurately determined using a gas metabolism analyzer (indirect calorimetry) to measure basal oxygen consumption and carbon dioxide production, followed by calculation via the Weir equation. Daily energy intake was strictly set to be isocaloric to each participant’s measured RMR (i.e., Energy Intake = Basal Energy Expenditure). This design ensured that the caloric intake was solely intended to meet the physiological requirements for maintaining basal life-sustaining activities. Standardized meals were prepared by professional dietitians according to the Chinese Food Composition Table (2018), with energy distribution across breakfast, lunch, and dinner at 30%, 40%, and 30%, respectively. The macronutrient composition was strictly maintained at 65% carbohydrates, 20% fats, and 15% proteins. Food selection prioritized low-glycemic index (GI) carbohydrates, high-quality proteins, and low animal fats, with cooking methods restricted to steaming, boiling, stewing, or cold dressing to strictly exclude deep-frying and high-oil preparation. Additionally, considering the physiological decline in RMR that typically occurs during weight loss, we performed weekly RMR re-assessments and dynamic dietary adjustments. Through this “weekly calibration,” we ensured that dietary intake consistently offset basal metabolic expenditure throughout the intervention period. In summary, as the dietary intake was designed to offset basal metabolism, the negative energy balance (caloric deficit) in this study was primarily driven by exercise-induced expenditure. This methodology ensures that the shifts in plasma metabolic profiles observed via metabolomics can be scientifically attributed to the biological response to the exercise load, rather than being a general byproduct of caloric deprivation.

### Clinical measures

All measurements were taken at baseline (pre-intervention) and on the morning following the conclusion of the intervention in a fasting state.

#### Body morphometry

Height (to the nearest 0.1 cm) and weight (to the nearest 0.01 kg) were measured using a calibrated stadiometer (RuKe, RK001) and an electronic scale (HuiBao, EB921), respectively. body mass index (BMI, kg/m^2^) was then calculated.

Chest, waist, and hip circumferences were measured using a non-elastic tape (WoLai, ER301) to the nearest 0.1 cm. Measurements were performed in triplicate, and the average value was recorded. The waist-to-hip ratio (WHR) was subsequently calculated.

#### Body composition

Body composition was assessed using a bioelectrical impedance analyzer (Inbody 370, Seoul, Korea). The test was conducted strictly according to standard operating procedures, requiring subjects to stand on the device in a standard posture after fasting and voiding their bladder. Bioimpedance data, including body fat percentage, fat mass, fat-free mass, total body water, and skeletal muscle mass, were recorded at frequencies of 5 kHz, 50 kHz, and 250 kHz.

#### Assessment of pubertal development

Pubertal status was assessed using the Self-Administered Rating Scale for Pubertal Development (PDS) (Chinese version) [20]. This scale has demonstrated high reliability and validity, with its classification results correlating strongly with clinical Tanner staging. Participants were categorized into five pubertal stages based on their total scores and physical milestones: Pre-puberty: (Females: score = 3, without menarche; Males: score = 3); Early Puberty: (Females: score = 4, without menarche; Males: score = 4 or 5, with no “3” responses); Mid-puberty: (Females: score = 5, without menarche; Males: score = 6–8, with no “4” responses); Late Puberty: (Females: score = 5, with menarche; Males: score = 9–11); Post-puberty: (Females: score = 12, with menarche; Males: score = 12).

#### Lipid metabolism and glucose homeostasis indicators

Fasting venous blood (5 mL) was collected from the antecubital vein into lithium heparin anticoagulant tubes. Samples were allowed to stand for 30 min and then centrifuged at 3000 rpm for 15 min at 4℃ to separate plasma. Plasma levels of total cholesterol (TC), triglycerides (TG), high-density lipoprotein cholesterol (HDL-C), and low-density lipoprotein cholesterol (LDL-C) were determined using an automatic biochemical analyzer.

Fasting blood glucose (FBG) was measured using biochemical analysis, and fasting insulin (FINS) was measured using electrochemiluminescence immunoassay. Insulin resistance index (HOMA-IR), insulin sensitivity index (HOMA-IS), and pancreatic β-cell function index (HOMA-β) were calculated using the homeostasis model assessment (HOMA) [21] as follows:

HOMA-IR = FBG(mmol/L) × FINS༈mIU/L༉/22.5;

HOMA-IS = 1/HOMA-IR;

HOMA-β = 20 × FINS(mIU/L)/(FBG༈mmol/L༉ − 3.5).

#### Statistical analysis

All statistical analyses were performed using R software (version 4.4.1). The normality of continuous variables was assessed using the Shapiro-Wilk test.

To evaluate the effect of the short-term Training Program intervention within each sex, clinical data for male and female subjects were compared pre- and post-intervention using paired sample t-tests (for normally distributed data) or Wilcoxon signed-rank tests (for non-normally distributed data). Normally distributed data are presented as Mean ± Standard Deviation (SD), while non-normally distributed data are presented as Median (First Quartile, Third Quartile) [Median (Q1, Q3)]. Additionally, a Chi-square test was performed to compare the distribution of pubertal staging between male and female participants.

To control for the influence of metabolic syndrome, insulin resistance, and inherent physiological sex differences, the Metabolic Syndrome Z-score (MetS Z-score) [22] and Pubertal staging were used as a covariate. Analysis of Covariance (ANCOVA) was employed to compare the relative change rates of clinical indicators between males and females, thereby assessing the degree of response to exercise intervention in obese adolescents of different sexes.

MetS Z_score=Z_WC + Z_MAP + Z_TG - Z_HDL + Z_HOMA_IR;

Relative Change Rate=[Pre-intervention value/(Pre-intervention value−Post-intervention value)]×100%.

All tests were two-tailed, with the significance level (α) set at 0.05. A value of *P <* 0.05 was considered statistically significant.

### Metabolomics analysis

#### Sample preparation

Preparation and analysis of plasma samples were conducted by Metabo-Profile Biotechnology Co., Ltd. (Shanghai). To minimize metabolite degradation, all samples were thawed on ice. A 20 µL plasma aliquot was transferred to a 96-well plate, and subsequent steps were performed using an Eppendorf epMotion workstation (Eppendorf Inc., Hamburg, Germany). First, 120µL of cold methanol containing internal standards was added to each well for protein precipitation, followed by vigorous vortexing for 5 min. The plate was then centrifuged at 4000 × g for 30 min at 4℃.

After centrifugation, the supernatant was aspirated, and 20 µL of freshly prepared derivatization reagent was added to each well. The plate was sealed and incubated at 30℃ for 60 min for derivatization. Following the reaction, 330µL of ice-cold 50% methanol solution was added to dilute the samples, followed by centrifugation at 4000 × g for 30 min at 4℃. Finally, 135µL of the supernatant was precisely transferred to a new 96-well plate, and 10µL of internal standard solution was added to each well. Calibration curves for quantitative analysis were prepared by serially diluting derivatized stock standards on the same plate. The plate was sealed for subsequent ultra-performance liquid chromatography-tandem mass spectrometry (UPLC-MS/MS) analysis.

#### UPLC-MS/MS analysis

Targeted metabolomics analysis of plasma samples was performed using an ACQUITY UPLC system coupled to a Xevo TQ-S tandem mass spectrometer (Waters Corp., Milford, MA, USA).

Chromatographic Separation: Samples were separated using an ACQUITY UPLC BEH C18 1.7 μm VanGuard pre-column (2.1 × 5 mm) and an ACQUITY UPLC BEH C18 1.7 μm analytical column (2.1 × 100 mm). The column temperature was maintained at 40 °C, and the sample manager was kept at 10℃. The mobile phases consisted of water with 0.1% formic acid (Phase A) and a mixture of acetonitrile and isopropanol (70:30, v/v) (Phase B). Gradient elution was performed according to a pre-programmed sequence to ensure effective separation of metabolites. The flow rate was set at 0.4 mL/min, and the injection volume was 5.0µL.

Mass Spectrometry: The mass spectrometer operated in both positive (ESI+) and negative (ESI-) electrospray ionization modes. The capillary voltage was set to 1.5 kV for positive mode and 2.0 kV for negative mode. The source temperature was 150℃, the desolvation temperature was 550 °C, and the desolvation gas flow was 1000 L/hr.

#### Data processing

The raw data generated by UPLC-MS/MS were processed according to a standardized workflow. First, a rigorous Quality Control (QC) system was implemented. QC samples, consisting of pooled plasma from all participants, were injected five times at the start of the analytical sequence for system equilibration and then interspersed every ten test samples to monitor instrument stability. In the data cleaning phase, unstable metabolites with a Relative Standard Deviation (RSD) > 30% in the QC samples were excluded [23]. To account for potential sexual dimorphism, a group-based filtering strategy was applied, retaining metabolites detected in ≥ 50% of samples within at least one sex group (male or female) [23]. Remaining missing values were filled using the minimum value imputation method [24]. Finally, signal drift and batch effects were corrected using a locally weighted regression algorithm based on the QC sample data to ensure robust comparability across the entire dataset.

#### Identification of sex-differential responsive metabolites

To accurately identify metabolites exhibiting sex-differential responses to the short-term Training Program intervention, Linear Mixed-Effects Models (LMM) were constructed using the lme4 and lmerTest packages in R. The dependent variable was the raw abundance of the metabolite. Fixed effects included Time, Sex, and their interaction (Time × Sex), with MetS Z-score included and Pubertal staging as a covariate. Subject ID was included as a random intercept to account for the non-independence of repeated measures within individuals. The model formula was: Metabolite_Abundance ~ Time * Sex + MetS Z_score + Pubertal staging + (1 | Subject_ID). Metabolites with a significant interaction term (*P <* 0.05) were identified as showing sex differences in response to short-term Training Program intervention.

#### Analysis and visualization of sex-differential responsive metabolites

To further elucidate the patterns of the sex-differential responses identified in Sect. "Identification of sex-differential responsive metabolites.Identification of sex-differential responsive metabolites", sex-stratified post-hoc analyses and visualizations were performed.

First, Principal Component Analysis (PCA) score plots were generated separately for male and female obese adolescents to evaluate overall separation trends before and after intervention.

Second, univariate statistical analyses were conducted separately for males and females to assess the effects of short-term Training Program within each sex.

Finally, volcano plots were drawn for males and females separately to visualize the comparison results, using thresholds of *P* < 0.05 and |log_2_FC|>0.

#### Identification of co-regulated metabolic modules

To investigate whether the Sex-differential Responsive Metabolites identified in Sect. "Identification of sex-differential responsive metabolites.Identification of sex-differential responsive metabolites" shared common regulatory patterns, hierarchical clustering analysis was performed.

First, the degree of response for each metabolite was calculated for every subject as the Log_2_ Fold Change [log_2_FC=log_2_(Post/Pre)]. Subsequently, to capture the core response trends for each sex, the mean log_2_FC of each metabolite was calculated separately for males and females, constructing a “Metabolite × Sex” response feature matrix.

To group metabolites based on similar sex-differential response patterns, hierarchical clustering was applied to the rows of this feature matrix. Prior to clustering, Z-score standardization was applied to eliminate differences in the magnitude of change between metabolites, ensuring clustering reflected only the similarity of response patterns. Clustering utilized Euclidean distance as the metric and the ward.D2 method for linkage. The dendrogram was cut to partition metabolites into co-regulated modules with distinct response patterns.

Finally, to visualize the actual direction and intensity of responses within each module, a heatmap was generated.

#### Functional annotation and pathway enrichment of co-regulated modules

To reveal the biological functions underlying each co-regulated module, pathway enrichment analysis was performed for each metabolite module identified in Sect. "Identification of co-regulated metabolic modules.Identification of co-regulated metabolic modules".

Metabolite lists from each module were submitted to the iMAP V1.0 online analysis platform (http://imap.metaboprofile.cloud). Analysis was conducted using the “KEGG Pathway Analysis” module with the species set to “Homo sapiens.” The enrichment analysis integrated Over-Representation Analysis (ORA) and pathway topology analysis. ORA calculated enrichment P-values based on the hypergeometric test to assess the enrichment of module metabolites in KEGG pathways. Pathway topology analysis evaluated the importance of metabolites within the pathway network structure using the “Out-degree Centrality” algorithm. Specifically, the Impact value represents the sum of the relative importance scores of the matched metabolites divided by the total importance scores of all metabolites in the given pathway [25].

Pathways with *P <* 0.05 and an Impact value > 0 [25]were considered significantly enriched in the co-regulated module, providing key clues for understanding the biological significance of sex-differential response patterns.

#### Identification of sex-differential metabolic biomarkers predicting response variables

To identify metabolic biomarkers capable of predicting changes in clinical indicators, Partial Least Squares Regression (PLS-R) models were constructed separately for males and females using Sex-differential Responsive Metabolites. This analysis was performed using the pls package in R.

Model Construction: For each sex-differential model, the predictor variables were the relative change rates of the Sex-differential Responsive Metabolites, and the response variables were the relative change rates of clinical indicators that showed significant differences between sexes. All predictor variables were centered and scaled to unit variance prior to model construction.

Model Optimization and Validation: To determine optimal model complexity and avoid overfitting, 10-fold cross-validation was used to select the best number of principal components. The optimal number of components was determined based on the minimum Root Mean Squared Error of Prediction (RMSEP) from cross-validation. Model performance was evaluated using the cumulative explained variance (R^2^Y) and the cumulative predictive ability (Q^2^) from cross-validation, where Q^2^ should be greater than 0. Prediction accuracy was categorized as small (Q^2^ >0), medium (Q^2^ >0.25), or large (Q^2^ >0.5) [26].

Screening of Key Predictors: For the final optimized models, the Variable Importance in Projection (VIP) score was calculated for each metabolite. Metabolites with a VIP > 1.0 were considered key biomarkers making significant contributions to the response variables. The direction of association between these biomarkers and the changes in response variables was determined by analyzing the regression coefficients of the model.

#### Functional annotation and pathway enrichment of predictive metabolic biomarkers

To uncover the underlying biological mechanisms driving changes in the response variables, the key predictive metabolites were compared and functionally annotated. First, a Venn diagram was used to identify the intersection and differences between the key predictive metabolites from the two PLS models, categorizing them into a shared subset and two sex-differential subsets. Subsequently, KEGG pathway enrichment analysis was performed separately for these three subsets. The analysis workflow and parameters were consistent with those described in Sect. "Functional annotation and pathway enrichment of co-regulated modules.Functional annotation and pathway enrichment of co-regulated modules".

## Results

### Effects of short-term training program intervention on body morphometry, body composition, lipid metabolism, and glucose homeostasis in obese adolescents of both sexes

The short-term training program intervention significantly improved multiple health indicators in obese adolescents. As shown in Table 1, after the 4-week standardized intervention, weight, BMI, body fat mass, body water, fat free mass, skeletal muscle mass and body fat percentage significantly decreased from baseline in both male and female obese adolescents (all *P <* 0.001). Specifically, the average weight decreased from 80.65 kg to 73.05 kg in males and from 81.75 kg to 71.85 kg in females. Furthermore, significant reductions were observed in chest circumference, waist circumference, and hip circumference, as well as the waist-to-hip ratio, in both sexes (all *P <* 0.001). These results indicate that the short-term training program intervention is effective in improving body morphometry and composition across both sexes.

**Table 1:** Effects of short-term training program intervention on body morphometry and body composition in obese adolescents of both sexes

Variables	Male(*n* = 58)		Female(*n* = 40)	
	Pre	Post	Pre	Post
Weight (kg)	80.65(72.90,97.80)	73.05(65.33,82.70)***	81.75(70.68,86.53)	71.85(63.78,79.00)***
BMI (kg/m^2^)	29.60(27.73,33.58)	25.95(24.40,29.48)***	30.25(28.05,32.10)	27.20(24.93,28.70)***
Chest circumference (cm)	101.3(95.1,107.8)	91.5(86.6,98.0)***	101.3(92.3,105.0)	94.0(85.0,99.1)***
Waist circumference (cm)	107.0(100.0,113.8)	96.8(90.3,103.8)***	99.8(91.5,108.3)	90.8(83.8,99.1)***
Hip circumference (cm)	107.0(100.0,114.5)	98.0(94.6,106.8)***	108.0(102.9,112.3)	101.5(96.4,105.2)***
Waist–hip ratio	0.97(0.94,1.01)	0.95(0.91,1.01)***	0.92(0.88,0.99)	0.91(0.85,0.97)***
Body water (L)	40.75(36.25,47.03)	38.85(34.40,43.25)***	36.90 ± 5.54	35.08 ± 5.34***
Fat free mass (kg)	56.60(50.38,65.33)	53.95(47.75,60.13)***	51.26 ± 7.70	48.72 ± 7.42***
Body fat mass (kg)	24.95(20.08,31.15)	18.90(14.65,25.18)***	28.45(22.60,32.73)	23.45(18.80,26.30)***
Skeletal muscle mass (kg)	52.05(46.00,60.08)	49.75(43.85,55.55)***	46.75 ± 6.87	44.62 ± 6.64***
Body fat percentage(%)	31.10(27.20,34.90)	25.25(22.38,29.68)***	35.60(33.18,37.70)	32.00(29.18,34.48)**

** denotes *P =* 0.001, and *** denotes *P <* 0.001

Further analysis of blood biochemical markers is presented in Table 2. Regarding lipid metabolism, TC, TG, and LDL-C levels significantly decreased from baseline in both sexes (all *P <* 0.001). Specifically, LDL-C levels decreased from 2.48 mmol/L to 1.68 mmol/L in males and from 2.48 mmol/L to 1.85 mmol/L in females. No statistically significant changes were observed in HDL-C before and after the intervention (*P >* 0.05).

In terms of glucose homeostasis, the short-term training program intervention exhibited sex-differential effects. In the female group, the HOMA-β index significantly decreased from 156.14 to 96.62 (*P =* 0.010), while no significant change was observed in the male group (*P =* 0.242). No significant differences were found in FBG, FINS, HOMA-IR or HOMA-IS in either sex (all *P >* 0.05). In summary, while the intervention effectively induced improvements in multiple core indicators, the differential response of HOMA-β between sexes suggests that the regulation of pancreatic β-cell function by short-term Training Program intervention may involve sex-distinct physiological pathways.

**Table 2:** Effects of short-term training program intervention on glucose homeostasis and lipid metabolism indicators in obese adolescents of both sexes

Variables	Male		Female	
	Pre	Post	Pre	Post
TC(mmol/L)	4.03(3.72,4.80)	3.23(2.95,3.80)^***^	4.17 ± 0.72	3.45 ± 0.58^***^
TG(mmol/L)	1.15(0.78,1.56)	0.63(0.50,0.80)^***^	1.09(0.79,1.27)	0.66(0.54,0.80)^***^
LDL-C(mmol/L)	2.48(2.13,3.00)	1.68(1.45,2.07)^***^	2.48 ± 0.53	1.85 ± 0.44^***^
HDL-C(mmol/L)	1.12(0.90,1.31)	1.13(1.00,1.25)	1.11 ± 0.20	1.12 ± 0.31
FINS(mIU/L)	8.42(6.72,12.91)	9.70(6.14,14.87)	11.20(9.00,14.27)	9.06(6.40,13.85)
FBG(mmol/L)	5.16(4.78,5.80)	5.01(4.58,5.33)	5.02(4.67,5.24)	5.31(4.73,5.81)
HOMA_β	108.69(68.05,175.52)	133.94(74.76,240.88)	156.14(102.71,258.70)	96.62(56.14,218.37)^**^
HOMA_IS	0.49(0.34,0.64)	0.44(0.30,0.77)	0.41(0.30,0.51)	0.44(0.31,0.66)
HOMA_IR	2.02(1.56,2.94)	2.27(1.30,3.37)	2.46(1.95,3.39)	2.28(1.52,3.25)

** denotes *P =* 0.001, and *** denotes *P <* 0.001

### Analysis of sex differences in the relative changes of metabolic indicators

Considering the potential influence of pubertal staging on the metabolic status and exercise stress response of adolescents, we first evaluated the distribution of developmental maturity between the two sexes using a Chi-square test. As shown in Table 3, a statistically significant difference was observed in the distribution of pubertal staging between males and females (χ^2^ = 18.775, *P <* 0.001). Overall, obese female adolescents exhibited a higher degree of developmental maturity compared to their male counterparts (85.0% of females were in Stage 5 of pubertal development, compared to 41.4% of males).

To further investigate the sex differences in response to the short-term training program intervention, we conducted an ANCOVA using the MetS Z-score and pubertal staging as covariates to compare the relative changes in clinical indicators between males and females. The results are presented in Table 4.

Regarding body morphometry and composition, after adjusting for developmental maturity and baseline metabolic risk, males exhibited a significantly greater magnitude of improvement compared to females in body fat mass reduction (23.49% vs. 17.62%, *P =* 0.003) and body fat percentage reduction (13.31% vs. 9.11%, *P =* 0.002). This confirms a sexual dimorphism in exercise-induced fat reduction, with males showing a distinct advantage. Additionally, the improvement in chest circumference was significantly greater in males than in females (8.29% vs. 6.36%, *P =* 0.031). However, no statistically significant differences were observed between sexes in the relative changes of weight, BMI, waist circumference, hip circumference, waist-hip ratio, skeletal muscle mass, body water, or fat free mass (all *P >* 0.05).

In terms of biochemical markers, sex differences were observed in glucose homeostasis regulation. The relative change in FBG differed significantly between sexes (*P =* 0.035), where males showed an improvement trend (percentage change of 4.93%), while females exhibited an upward trend (percentage change of −6.58%). Similarly, the change in HOMA-β differed significantly between sexes (*P =* 0.031), with females exhibiting a more pronounced downregulation following the intervention. Regarding lipid metabolism, although both sexes showed significant internal improvements as described in Sect. "Effects of short-term training program intervention on body morphometry, body composition, lipid metabolism, and glucose homeostasis in obese adolescents of both sexes", there were no statistically significant differences in the magnitude of improvement for TC, TG, LDL-C, or HDL-C between the two groups after adjusting for pubertal stage (all *P >* 0.05).

These ANCOVA-based findings confirm that even after accounting for the influence of developmental maturity and baseline metabolic syndrome risk, significant sex differences exist in the magnitude of fat reduction and glucose homeostasis regulation. These results provide a robust foundation for the subsequent sex-stratified metabolomics analysis to explore the underlying molecular regulatory mechanisms.

**Table 3:** Sex differences in pubertal staging among obese adolescents

Pubertal staging	Sex		χ²	*P*
	Male(*n* = 58)	Female(*n* = 40)		
Stage 3, n(%)	19(32.8%)	4(10.0%)	18.775	< 0.001
Stage 4, n(%)	15(25.9%)	2(5.0%)		
Stage 5, n(%)	24(41.4%)	34(85.0%)		

**Table 4:** Comparison of relative changes of indicators between sexes following short-term training program intervention

Relative change (%)	Male	Female	*P*
Weight	11.33 ± 3.13	9.86 ± 2.71	0.174
BMI	10.82(8.92,13.36)	9.59(7.96,11.14)	0.247
Chest circumferences	8.29(5.87,11.65)	6.36(4.92,9.18)	0.031
Waist circumference	8.30(6.28,12.03)	6.83(5.33,9.07)	0.447
Hip circumference	6.53(4.95,8.85)	6.29(4.67,7.17)	0.307
Waist–hip ratio	1.62(0.51,4.57)	1.26(−0.21,3.42)	0.990
Body fat mass	23.49(19.20,29.26)	17.62(14.03,22.58)	0.003
Skeletal muscle mass	4.64(3.60,6.38)	4.32(3.31,6.10)	0.790
Body water	5.05(3.97,6.69)	4.79(3.66,6.63)	0.697
Fat free mass	5.10(3.93,6.69)	4.85(3.54,6.68)	0.673
Body fat percentage	13.31(10.26,19.16)	9.11(6.67,12.71)	0.002
TC	19.95 ± 12.60	15.96 ± 15.34	0.638
TG	46.57(18.95,59.48)	40.15(6.40,49.05)	0.725
LDL-C	30.96(22.94,40.81)	26.84(14.39,35.08)	0.215
HDL-C	−0.97(−15.42,10.92)	−1.21(−16.40,10.19)	0.595
FINS	1.35(−19.63,24.95)	27.55(−5.20,40.06)	0.195
FBG	4.93(−9.45,14.03)	−6.58(−17.69,6.79)	0.035
HOMA_β	−6.41(−81.77,36.50)	32.86(−3.71,64.31)	0.031
HOMA_IS	−1.24(−35.78,20.50)	−32.44(−74.70,26.45)	0.615
HOMA_IR	1.21(−25.80,26.31)	24.49(−36.57,42.74)	0.557

Relative changes (%) = (Pre-intervention value - Post-intervention value)/Pre-intervention value × 100%

### Sex-differential responsive metabolites to short-term training program intervention in obese adolescents

Targeted metabolomics analysis was performed on plasma samples from obese adolescents before and after short-term Training Program intervention, successfully detecting 204 plasma metabolites covering 16 categories, including fatty acids, amino acids, organic acids, etc.

To identify plasma metabolites exhibiting sex-differential responses to short-term Training Program intervention, LMM were utilized. As shown in Table 5, LMM analysis identified 65 sex-differential Responsive Metabolites showing significant sex-by-time interaction effects post-intervention (*P* < 0.05). These metabolites spanned multiple metabolic categories, including fatty acids, amino acids, organic acids, acylcarnitines, etc., providing a metabolic basis for further revealing the sex-differentiated mechanisms of exercise-induced improvements in metabolic health.

**Table 5:** Sex-differentiated responsive metabolites in obese adolescents under short-term training program intervention

Metabolite	Class	Male_Pre	Male_Post	Female_Pre	Female_Post	*P*
Alanine	Amino Acids	371.56 ± 83.85	276.03 ± 47.74	355.76 ± 81.59	312.00 ± 62.16	0.004
Asparagine	Amino Acids	38.32 ± 7.25	39.19 ± 7.15	38.32 ± 9.29	42.97 ± 9.44	0.003
Aspartic acid	Amino Acids	3.67 ± 1.50	1.87 ± 0.73	2.79 ± 1.75	1.72 ± 0.61	0.033
Citrulline	Amino Acids	23.30 ± 4.69	19.39 ± 3.66	22.81 ± 4.08	21.37 ± 4.45	0.002
Creatine	Amino Acids	35.73 ± 10.85	17.30 ± 5.65	26.49 ± 10.35	16.77 ± 8.52	< 0.001
Cystine	Amino Acids	84.35 ± 46.62	76.20 ± 28.75	72.67 ± 39.84	84.61 ± 39.06	0.026
Glutamic acid	Amino Acids	51.12 ± 19.73	31.04 ± 7.15	41.03 ± 12.78	28.30 ± 5.31	0.038
Isoleucine	Amino Acids	75.74 ± 29.00	93.42 ± 26.30	82.89 ± 31.81	82.68 ± 25.87	0.017
Methionine	Amino Acids	48.35 ± 7.91	45.22 ± 7.13	47.10 ± 6.93	48.08 ± 6.31	0.014
N-Acetylaspartic acid	Amino Acids	0.36 ± 0.08	0.38 ± 0.08	0.34 ± 0.06	0.41 ± 0.09	0.020
Ornithine	Amino Acids	24.51 ± 5.48	24.52 ± 4.64	24.27 ± 6.49	26.98 ± 5.37	0.024
Phenylalanine	Amino Acids	65.56 ± 15.56	48.55 ± 10.05	56.19 ± 13.24	48.16 ± 12.75	0.005
Pipecolic acid	Amino Acids	5.32 ± 2.01	8.60 ± 2.22	4.93 ± 1.61	7.06 ± 1.72	0.018
Pyroglutamic acid	Amino Acids	57.06 ± 23.28	33.56 ± 7.63	45.13 ± 14.68	31.24 ± 5.44	0.020
Serine	Amino Acids	129.33 ± 24.43	163.24 ± 27.15	135.92 ± 26.56	183.97 ± 30.88	0.007
Threonine	Amino Acids	54.87 ± 10.79	55.93 ± 10.14	56.02 ± 10.94	64.70 ± 12.29	0.004
Tryptophan	Amino Acids	76.34 ± 14.23	54.69 ± 7.82	67.54 ± 13.22	56.25 ± 11.10	< 0.001
Tyrosine	Amino Acids	75.50 ± 13.87	54.09 ± 7.93	71.76 ± 12.68	57.78 ± 8.88	0.007
beta-Alanine	Amino Acids	4.47 ± 1.09	4.68 ± 1.13	4.13 ± 1.16	4.75 ± 1.20	0.031
Benzenebutanoic acid	Benzenoids	0.11 ± 0.02	0.10 ± 0.02	0.10 ± 0.01	0.10 ± 0.02	0.013
Phenylpyruvic acid	Benzenoids	5.66 ± 1.76	3.41 ± 0.94	4.28 ± 1.09	3.57 ± 0.98	< 0.001
4-Hydroxybenzoic acid	Benzoic Acids	0.42 ± 0.03	0.43 ± 0.04	0.47 ± 0.19	0.43 ± 0.03	0.040
Gallic acid	Benzoic Acids	0.74 ± 0.64	0.81 ± 0.59	1.12 ± 0.71	0.78 ± 0.60	0.010
Glyceric acid	Carbohydrates	4.41 ± 0.81	3.57 ± 0.42	3.76 ± 0.96	3.30 ± 0.53	0.015
Threonic acid	Carbohydrates	1.39 ± 0.62	1.08 ± 0.31	1.20 ± 0.48	1.12 ± 0.34	0.030
Xylose	Carbohydrates	3.28 ± 0.94	2.55 ± 0.63	2.76 ± 0.79	2.63 ± 0.53	0.001
2-Methylbutyroylcarnitine(C5: 0)	Carnitines	0.12 ± 0.04	0.09 ± 0.02	0.10 ± 0.03	0.09 ± 0.02	0.002
Acetylcarnitine(C2: 0)	Carnitines	70.47 ± 26.94	93.78 ± 26.80	65.29 ± 23.46	74.01 ± 23.53	0.028
Adipoylcarnitine(C6: 0-DC)	Carnitines	0.12 ± 0.06	0.14 ± 0.05	0.13 ± 0.05	0.12 ± 0.04	0.002
Carnitine(C0)	Carnitines	44.75 ± 9.53	33.00 ± 8.26	36.77 ± 12.03	35.37 ± 11.62	< 0.001
Decanoylcarnitine(C10: 0)	Carnitines	1.00 ± 0.33	0.91 ± 0.30	0.99 ± 0.55	0.75 ± 0.28	0.046
Glutarylcarnitine(C5: 0-DC)	Carnitines	0.14 ± 0.04	0.11 ± 0.03	0.13 ± 0.03	0.11 ± 0.02	0.008
Isovalerylcarnitine(C5: 0)	Carnitines	0.35 ± 0.12	0.20 ± 0.11	0.23 ± 0.08	0.18 ± 0.09	< 0.001
Oleylcarnitine(C18: 1)	Carnitines	5.90 ± 1.92	8.57 ± 1.78	5.76 ± 1.31	7.27 ± 1.45	0.001
Palmitoylcarnitine(C16: 0)	Carnitines	3.05 ± 0.67	3.77 ± 0.64	2.91 ± 0.46	3.35 ± 0.54	0.023
Propionylcarnitine(C3: 0)	Carnitines	1.05 ± 0.29	0.53 ± 0.12	0.78 ± 0.31	0.55 ± 0.21	< 0.001
10Z-Heptadecenoic acid	Fatty Acids	2.80 ± 1.21	3.47 ± 1.30	3.00 ± 1.29	2.90 ± 1.21	0.005
10Z-Nonadecenoic acid	Fatty Acids	1.53 ± 0.54	2.05 ± 0.55	1.56 ± 0.55	1.72 ± 0.58	0.002
2-Hydroxy-3-methylbutyric acid	Fatty Acids	17.95 ± 8.25	34.09 ± 18.81	12.86 ± 3.99	20.11 ± 7.64	< 0.001
9E-tetradecenoic acid	Fatty Acids	0.70 ± 0.26	0.73 ± 0.20	0.80 ± 0.62	0.68 ± 0.46	0.007
Adrenic acid	Fatty Acids	6.86 ± 2.12	9.53 ± 2.41	6.00 ± 1.73	7.23 ± 2.35	0.007
Arachidonic acid	Fatty Acids	103.43 ± 29.76	129.58 ± 27.52	95.09 ± 29.55	105.23 ± 33.44	0.012
Azelaic acid	Fatty Acids	0.13 ± 0.04	0.09 ± 0.03	0.11 ± 0.04	0.09 ± 0.04	0.020
DHA	Fatty Acids	58.26 ± 22.89	59.36 ± 21.65	56.95 ± 24.73	49.92 ± 18.96	0.026
DPA	Fatty Acids	4.50 ± 1.52	5.41 ± 1.78	4.31 ± 1.68	4.38 ± 1.67	0.015
DPAn-6	Fatty Acids	2.46 ± 0.86	3.04 ± 0.87	2.13 ± 0.71	2.31 ± 0.91	0.012
Linoelaidic acid	Fatty Acids	5.03 ± 1.30	6.00 ± 1.27	5.42 ± 1.45	5.35 ± 1.63	0.002
Linoleic acid	Fatty Acids	260.04 ± 58.32	286.36 ± 51.22	265.94 ± 62.32	253.75 ± 69.17	0.008
Methylglutaric acid	Fatty Acids	0.14 ± 0.08	0.12 ± 0.06	0.13 ± 0.09	0.15 ± 0.09	0.021
Myristic acid	Fatty Acids	36.36 ± 10.43	37.18 ± 10.35	40.15 ± 12.36	33.51 ± 10.32	0.004
Myristoleic acid	Fatty Acids	2.36 ± 1.17	2.90 ± 1.19	2.65 ± 1.30	2.65 ± 1.37	0.026
Oleic acid	Fatty Acids	860.45 ± 182.54	1028.10 ± 164.97	863.71 ± 170.96	899.48 ± 214.13	0.007
Palmitoleic acid	Fatty Acids	32.73 ± 11.99	40.94 ± 12.34	35.12 ± 12.44	34.85 ± 12.78	0.004
Undecylenic acid	Fatty Acids	1.04 ± 0.72	0.77 ± 0.58	0.85 ± 0.69	0.86 ± 0.61	0.013
alpha-Linolenic acid	Fatty Acids	20.92 ± 8.66	22.16 ± 7.99	21.10 ± 8.90	18.98 ± 8.59	0.041
Indole-3-carboxylic acid	Indoles	0.11 ± 0.05	0.13 ± 0.05	0.13 ± 0.05	0.12 ± 0.04	0.015
2-Hydroxybutyric acid	Organic Acids	161.67 ± 90.79	230.35 ± 59.11	153.18 ± 83.24	160.42 ± 59.22	0.001
3-Hydroxybutyric acid	Organic Acids	124.75 ± 138.81	356.24 ± 127.75	148.03 ± 147.44	220.59 ± 137.23	< 0.001
Glycolic acid	Organic Acids	7.73 ± 2.47	7.45 ± 1.97	6.50 ± 2.47	7.40 ± 2.25	0.016
Lactic acid	Organic Acids	1379.27 ± 371.60	1303.44 ± 188.14	1164.44 ± 246.43	1292.88 ± 201.43	0.002
Methylmalonic acid	Organic Acids	3.03 ± 0.41	3.16 ± 0.44	2.79 ± 0.41	3.15 ± 0.51	0.023
Oxoglutaric acid	Organic Acids	87.80 ± 36.42	69.32 ± 12.11	64.69 ± 16.58	63.40 ± 10.58	0.008
Succinic acid	Organic Acids	2.99 ± 0.44	3.15 ± 0.50	2.76 ± 0.47	3.14 ± 0.55	0.042
4-Hydroxyphenylpyruvic acid	Phenols	6.84 ± 1.93	4.40 ± 1.02	5.91 ± 1.82	4.35 ± 1.08	0.031
Butyric acid	SCFAs	2.40 ± 0.50	1.95 ± 0.42	2.27 ± 0.54	2.08 ± 0.34	0.002

### Response patterns of sex-differential responsive metabolites

**Fig. 1:**
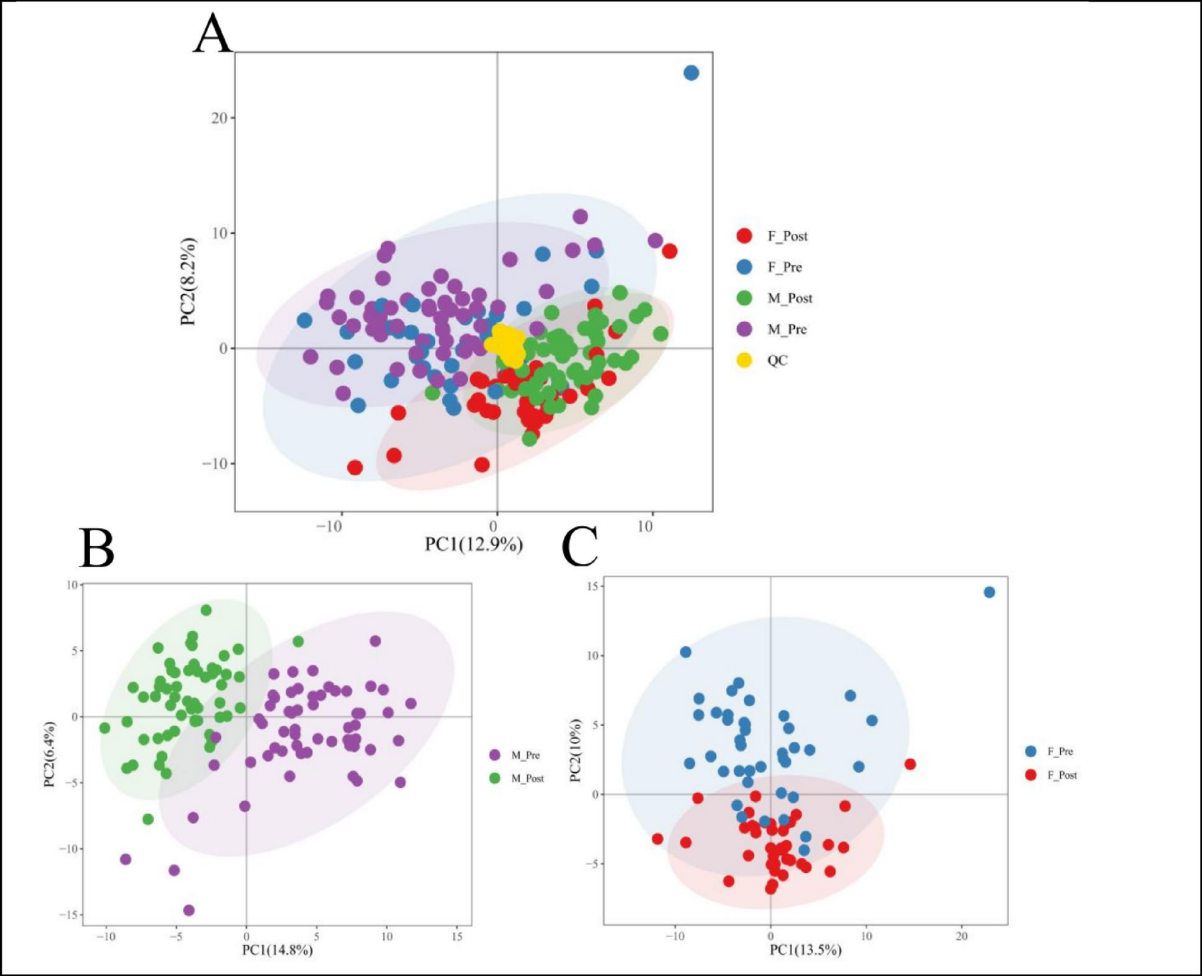
PCA of short-term Training Program Intervention on Obese Adolescents. (**A**) Unsupervised PCA score plot of all plasma samples, including males (M) and females (F) before (Pre) and after (Post) the 4-week short-term Training Program intervention. (**B**) Metabolic shift analysis of male metabolites before and after short-term Training Program intervention; (**C**) Metabolic shift analysis of female metabolites before and after short-term Training Program intervention;

To comprehensively evaluate the metabolic effects of the short-term training program intervention across sexes, we first examined the metabolic profiles of all samples using global PCA (Fig. 1A). The QC samples were tightly clustered, confirming the stability of the analytical system. Along the PC1 and PC2 axes, male and female samples showed significant clustering separation at baseline (Pre-intervention), demonstrating a strong baseline sexual dimorphism in the metabolic profiles of obese adolescents. Following the intervention (Post-intervention), the metabolic trajectories of the two sexes exhibited distinct directional shifts, suggesting that exercise-induced metabolic remodeling is sex-differential.

**Fig. 2:**
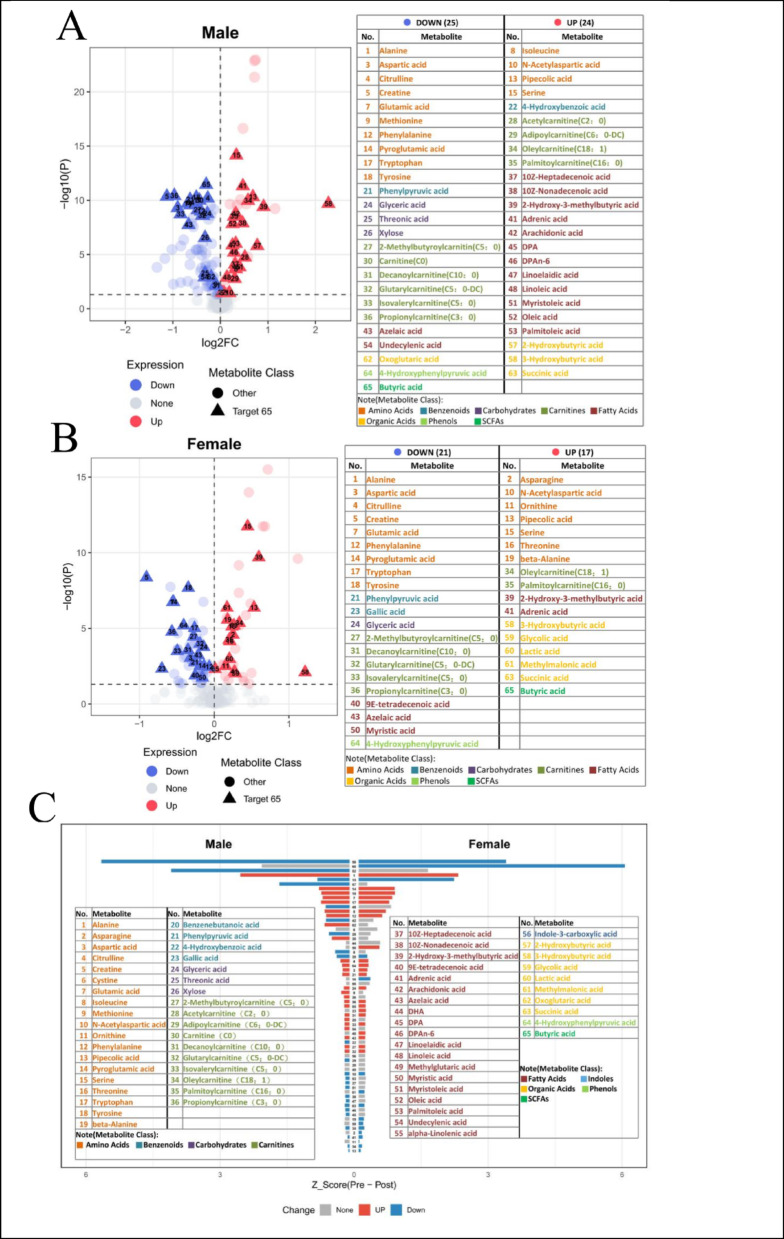
Metabolic Impact of short-term Training Program Intervention on Obese Adolescents of Different Sexes. (**A-B**) Volcano plots of differential metabolites in males (**A**) and females (**B**) before and after short-term Training Program intervention. In these plots, the x-axis represents the Log_2_FC, and the y-axis represents the negative logarithm of the P-value (− log_10_P). Red dots indicate significantly upregulated metabolites, blue dots indicate significantly downregulated metabolites (*P <* 0.05), and gray dots indicate no significant difference. Triangles denote Sex-differential Responsive Metabolites identified by Linear Mixed-Effects Models. (**C**) Changes of Sex-differential Responsive Metabolites in both sexes

Intra-group differential analysis was further conducted to observe the respective metabolic responses of each sex (Figs. 1B-C and 2A-B). Of the 204 detected metabolites, 115 (56.4% of the total) changed significantly in males (40 upregulated, 75 downregulated), while 94 (46.1% of the total) changed significantly in females (38 upregulated, 56 downregulated). Among the 65 sex-differential responsive metabolites identified via significant “Time × Sex” interactions, 49 (75.4%) showed significant intra-group differences in males, with a balanced ratio of upregulated (24) to downregulated (25) metabolites. In contrast, only 38 (58.5%) of these metabolites showed significant intra-group differences in females, with 21 exhibiting a downward trend.

Volcano plots (Fig. 2A-B) visually display the metabolic markers most sensitive to the training program. In males, the most significantly upregulated metabolite was 3-hydroxybutyrate (No. 58), which exhibited the largest fold change and high statistical significance. Additionally, serine (No. 15), adrenic acid (No. 41, C22:4), and various long-chain acylcarnitines, such as oleoylcarnitine (No. 34, C18:1) and palmitoylcarnitine (No. 35, C16:0), showed significant upregulation. The most notable decreases in males were observed for creatine (No. 5) and propionylcarnitine (No. 36, C3:0). In females, the response pattern was markedly different. The most significantly upregulated metabolites were serine (No. 15) and 2-hydroxy-3-methylbutyric acid (No. 39), with asparagine (No. 2) also increasing significantly. Conversely, creatine (No. 5), tyrosine (No. 18), and pyroglutamic acid (No. 14) exhibited significant downward trends in females. The differential distribution of these sex-differential Responsive Metabolites further confirms the significant sexual dimorphism in exercise-induced metabolic remodeling.

Visual analysis of the response intensity of the 65 sex-differential Responsive Metabolites (Fig. 2C) revealed similarities and differences in molecular pathways. Some metabolites showed consistent directional responses; for example, 2-hydroxy-3-methylbutyric acid, 3-hydroxybutyric acid, and adrenic acid significantly increased in both sexes, while 2-methylbutyrylcarnitine (C5:0 carnitine), 4-hydroxyphenylpyruvic acid, and alanine significantly decreased. However, more pronounced differences were observed in categories: multiple unsaturated fatty acids (e.g., 10Z-heptadecenoic acid) and acetylcarnitine (C2:0) were significantly upregulated in males (but did not reach significance in females), whereas asparagine, β-alanine, and threonine showed significant upward trends in females (but did not reach significance in males).

### Co-regulation modules and pathway analysis of sex-differential responsive metabolites

**Fig. 3:**
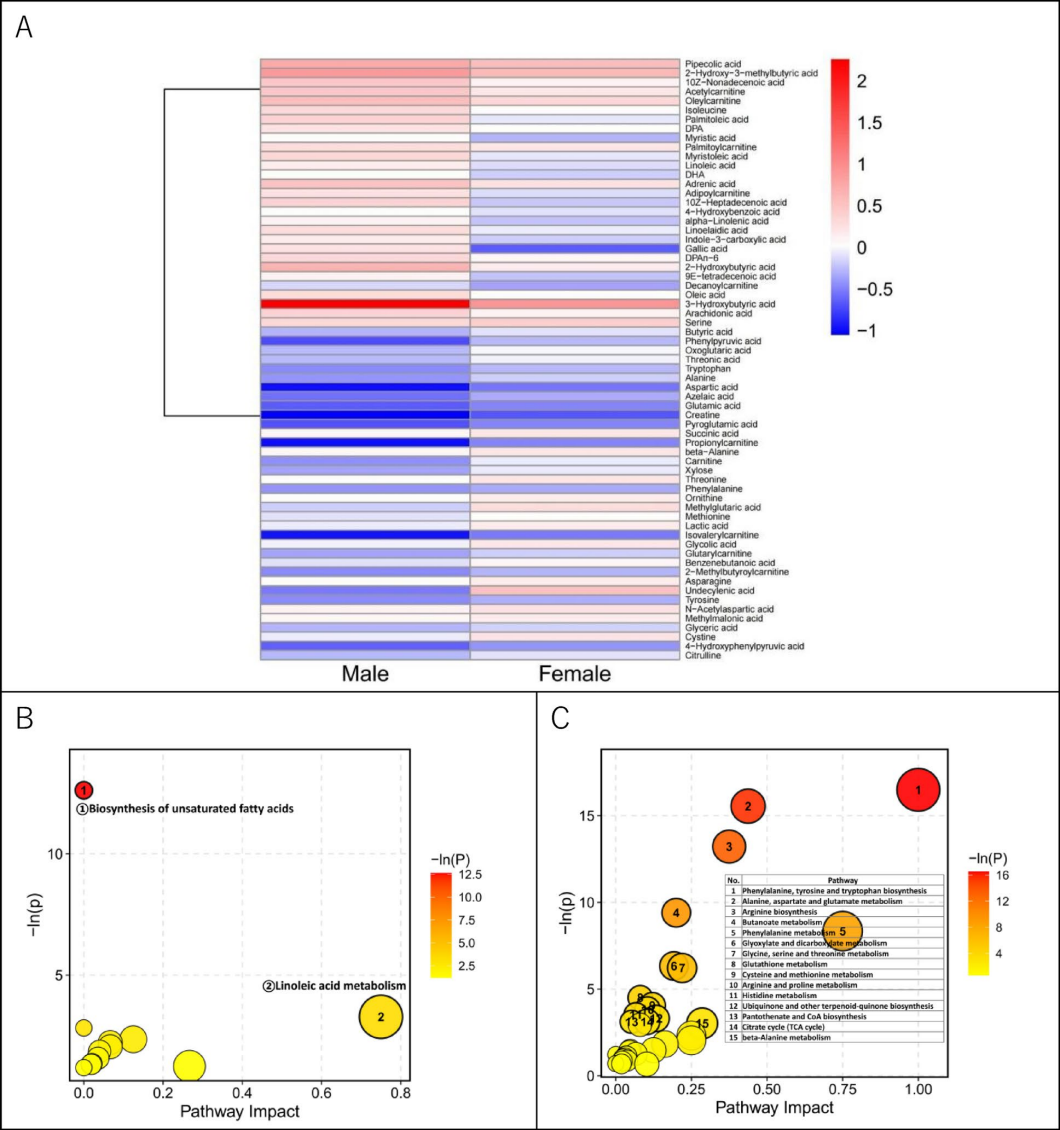
Hierarchical Clustering analysis and Pathway Analysis of Sex-differential Responsive Metabolites. (**A**) Hierarchical clustering analysis of Sex-differential Responsive Metabolites. These metabolites were identified via LMM with a significant “Time × Sex” interaction (*P <* 0.05). Rows represent individual metabolites arranged by clustering order, and columns represent the Male and Female groups. The color scale represents the Z-score standardized mean log_2_ Fold Change (log_2_ FC = log_2_ (Post/Pre), where red indicates upregulation and blue indicates downregulation following the intervention. (**B-C**) Pathway analysis of (**B**) Module 1 metabolites and (**C**) Module 2 metabolites. The y-axis denotes -In(P) values from over-representation analysis, and the x-axis denotes the Pathway Impact values from topology analysis. Bubbles are explicitly labeled for pathways reaching statistical significance (*P <* 0.05)

To further analyze the regulatory patterns and biological functions of the 65 sex-differential responsive metabolites, hierarchical clustering analysis was performed (Fig. 3A). Based on the mean log_2_FC response values between sexes, two metabolic modules with distinct response characteristics were identified.

Cluster 1 contained 28 metabolites, primarily composed of unsaturated fatty acids (e.g., linoleic acid, α-linolenic acid, and arachidonic acid) and acylcarnitines. Pathway enrichment analysis of this module (Fig. 3B) revealed significant enrichment in the linoleic acid metabolism pathway. This module exhibited distinct sex differences, showing a clear upward trend in males (mean log_2_FC = 0.385) but a minimal response in females (mean log_2_FC = 0.028). This suggests that under the same training intensity, the linoleic acid metabolism pathway is more robustly activated in males than in females.

Cluster 2 contained 37 metabolites, mainly consisting of amino acids, organic acids, and short-chain acylcarnitines. Pathway enrichment analysis (Fig. 3C) showed that these metabolites were primarily involved in the citrate cycle (TCA cycle) and 15 amino acid metabolism-related pathways, including phenylalanine, glycine, serine, and threonine metabolism. This module showed a downward trend in both sexes; however, the magnitude of downregulation was significantly greater in males (log_2_FC = −0.333) than in females (log_2_FC = −0.077). This indicates that while the training program induced a shared regulatory direction in amino acid and energy metabolism pathways across both sexes, the stronger response magnitude in males resulted in more statistically significant enrichment features.

In summary, the results suggest that both sexes share a similar regulatory direction (downregulation) in amino acid and energy metabolism pathways, though they differ in the magnitude of change. Furthermore, males exhibited a distinct trend in the activation of fatty acid metabolism pathways.

### Correlation analysis between sex-differential responsive metabolites and clinical indicators

To explore the predictive capacity of the 65 sex-differential responsive metabolites for the improvement of clinical indicators, this study combined the sex-differential responsive indicators identified in Sect. "Analysis of sex differences in the relative changes of metabolic indicators" (such as FBG) and evaluation indicators related to metabolic health (such as body weight), and constructed separate PLS models for males and females (Table 6). The results revealed that the predictive performance of metabolites for clinical improvements exhibited sex differences.

In females, the sex-differential responsive metabolites demonstrated predictive capacity for metabolic improvements. The PLS models exhibited moderate predictive power for LDL-C (R^2^Y = 0.3895, Q^2^ = 0.1853) and FBG (R^2^Y = 0.3552, Q^2^ = 0.1321). However, the models showed limited predictive capacity for weight, chest circumference, body fat mass, body fat percentage, and HOMA-β (Q^2^ < 0). In males, despite their superior improvements in body composition as shown in Sect. "Analysis of sex differences in the relative changes of metabolic indicators", the sex-differential metabolites generally showed insufficient predictive capacity for clinical changes. The PLS model only explained a portion of the variance for FBG (R^2^Y = 0.2653), but its predictive power was weak (Q^2^ = 0.0091). No effective predictive capacity was observed for weight, chest circumference, fat mass, body fat percentage, LDL-C, or HOMA-β in males (Q^2^ < 0).

**Fig. 4:**
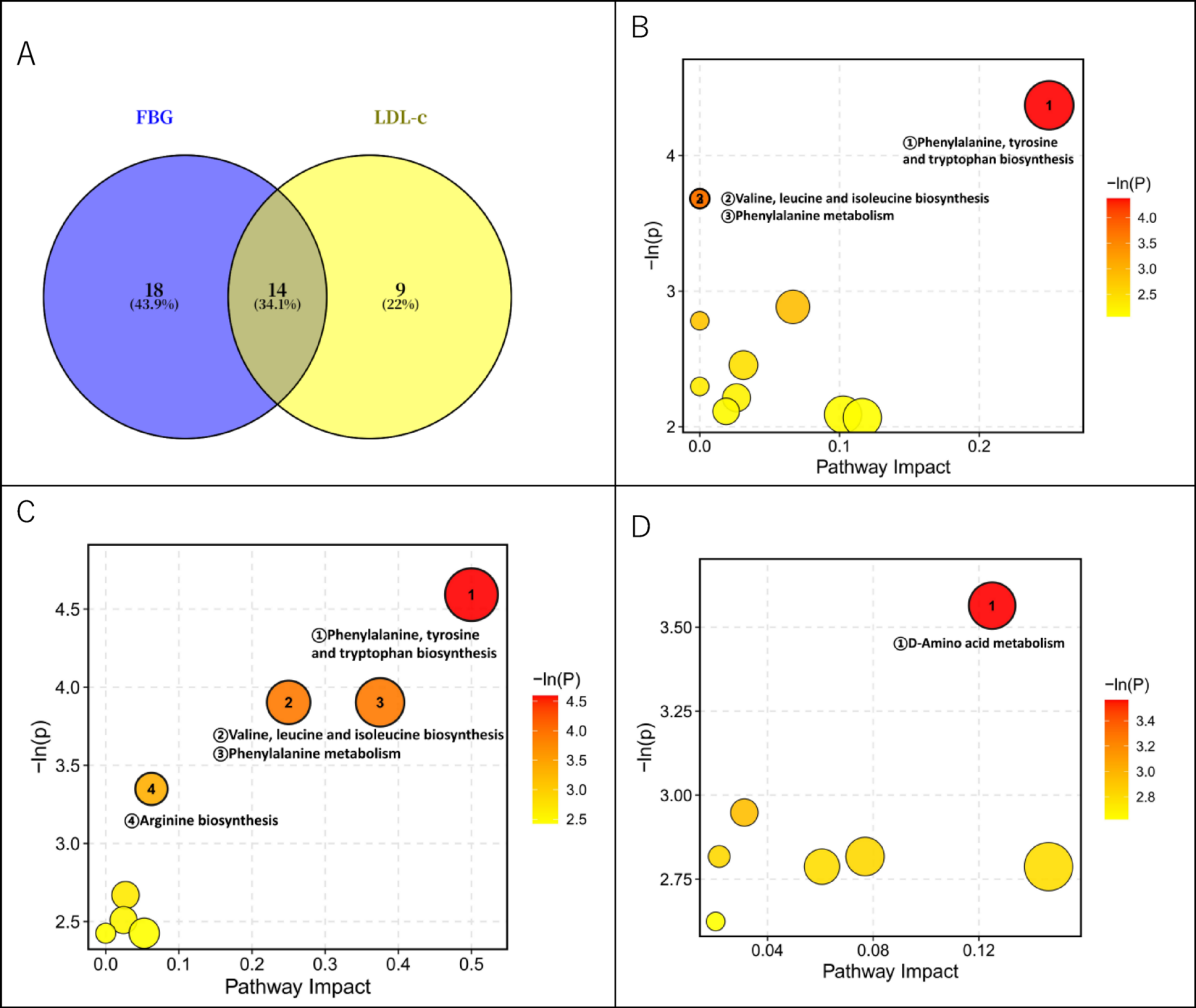
Pathway Analysis of Sex-differential Responsive Metabolites Predicting Female FBG and LDL-C. (**A**) Venn diagram of metabolites capable of predicting female FBG and LDL-C; (**B**) Pathway analysis of shared metabolites; (**C**) Pathway analysis of metabolites associated with FBG prediction in females; (**D**) Pathway analysis of Metabolites associated with LDL-c prediction in females

For the metabolites predicting FBG and LDL-C in females, we further analyzed their overlap using a Venn diagram (Fig. 4A). The analysis showed that 14 metabolites were shared predictors for both FBG and LDL-C in females, while 18 metabolites were uniquely associated with FBG prediction and 9 metabolites were uniquely associated with LDL-C prediction. Pathway enrichment analysis revealed that the shared predictive metabolites were primarily enriched in the phenylalanine, tyrosine, and tryptophan biosynthesis pathway (Fig. 4B). Metabolites associated with FBG prediction in females involved a broader amino acid network, including phenylalanine, tyrosine, and tryptophan biosynthesis; valine, leucine, and isoleucine biosynthesis; phenylalanine metabolism; and arginine biosynthesis (Fig. 4C). Conversely, metabolites associated with LDL-C prediction in females were linked to the D-amino acid metabolism pathway (Fig. 4D).

**Table 6:** Robustness of constructed PLS models

Weight	*R*^2^Y-Male	*R*^2^Y-Female	Q^2^-Male	Q^2^-Female
	0.3871	0.3583	−0.7749	−0.0076
Fat Mass	0.3814	0.2826	−0.9746	−0.0529
FBG	0.2653	0.3552	0.0091	0.1321
LDL-c	0.1881	0.3895	−0.1080	0.1853
Chest Circumference	0.3736	0.3967	−0.7025	−0.3463
Body Fat Percentage	0.2866	0.2150	−0.7450	−0.1375
HOMA-β	0.2279	0.4290	−1.2926	−0.4148

## Discussion

Through a four-week, fully supervised, and standardized training program, this study systematically explored the sexual dimorphism in metabolic responses to short-term Training Program in obese adolescents using a metabolomics approach. Clinical outcomes indicated that the intervention primarily induced fat reduction in males, whereas females exhibited enhanced glucose homeostasis. Further molecular analysis revealed that males appear to leverage a lipid-oxidation/protein-sparing synergy, while females prioritize amino-acid network fine-tuning. Based on these core findings, this study provides a progressive analysis ranging from clinical phenotypic differentiation to molecular mechanism elucidation and predictive modeling.

### Sex differences in fat reduction and glucolipid improvement under short-term training program intervention

This study found that a 4-week standardized training program yielded significant health benefits for obese adolescents of both sexes (Tables 1 and 2). However, after adjusting for pubertal staging and baseline metabolic risk, we observed that the magnitude and pattern of these responses were sex-differential.

First, regarding body composition, males demonstrated a significantly greater advantage in fat reduction. Although no statistical difference was observed between sexes in the improvement of weight or BMI (*P >* 0.05), the reductions in fat mass (−23.49% vs. −17.62%, *P =* 0.003) and body fat percentage (−13.31% vs. −9.11%, *P =* 0.002) were significantly superior in males. This dimorphism likely results from the synergy between the endocrine environment and adipose tissue characteristics. The relatively higher testosterone levels in males promote lipolysis and help maintain muscle mass, providing a favorable metabolic environment for sustained fatty acid oxidation [27]. Furthermore, the typical visceral fat distribution in males [28] exhibits higher biological sensitivity to lipolytic hormones, such as catecholamines, released during exercise [29], making it a more readily mobilized energy reservoir. In explaining these differences, it is also essential to distinguish between acute metabolic effects and long-term adaptive effects. While previous research suggests that females may exhibit stronger lipid mobilization or oxidation during a single acute bout of exercise [30], the overall fat-loss response in females over this 4-week intervention was somewhat delayed compared to males. This phenotypic difference may reflect an adaptive strategy where estrogen in females prioritizes the preservation of subcutaneous fat (particularly in the gluteofemoral region) to maintain the energy homeostasis required for reproductive potential [31].

Second, regarding glucose homeostasis, females exhibited more refined optimization. HOMA-β reflects the basal secretory function of pancreatic β-cells; its decrease, in the context of stable blood glucose, serves as indirect evidence of improved insulin sensitivity and reduced secretory load [32]. We observed a significant reduction in HOMA-β in females (*P =* 0.010), which was significantly greater than that in males (*P =* 0.031). This suggests that under the same exercise load, females may maintain glucose homeostasis by enhancing insulin efficiency. This may be linked to unique physiological mechanisms: estrogen exerts protective and regulatory effects on insulin signaling pathways and β-cell function [33], and short-term Training Program may further optimize tissue-level communication by improving the metabolic health of subcutaneous adipose tissue in females [34]. The significant difference in the relative change of fasting blood glucose (FBG) between sexes (*P =* 0.035) further supports this optimized glucose management profile in females.

Finally, concerning lipid metabolism, while both sexes showed significant internal improvements, the magnitude of change (TC, TG, LDL-C) did not reach statistical significance between groups after adjusting for covariates (all *P >* 0.05). Combined with our omics results, the lipid improvements in males were accompanied by significantly greater fat loss and acylcarnitine fluctuations compared to females, suggesting that male lipid benefits rely more on systemic fatty acid mobilization and increased oxidative flux [35]. In contrast, female participants achieved comparable lipid improvements despite a slower rate of fat loss. This suggests that females may prioritize the fine-tuning of amino acid metabolism networks to optimize insulin sensitivity, thereby achieving metabolic benefits through enhanced lipid uptake or suppressed endogenous synthesis without relying on large-scale adipose depletion. This finding aligns with cross-species studies on white adipose tissue, which have observed that males exhibit stronger lipolysis and oxidative signaling under exercise stress, whereas females show more prominent glucose signaling characteristics [36].

### Males favor fatty acid oxidation and protein-sparing strategies during short-term training program

Our metabolomics results suggest that obese male adolescents adopt a metabolic strategy characterized by full-chain fatty acid oxidation coupled with protein-sparing, consistent with their significant fat-loss outcomes.

Males demonstrated a full-chain activation of fatty acid oxidation. First, the intervention triggered peripheral lipid mobilization, evidenced by the upregulation of various unsaturated fatty acids (e.g., linoleic acid, 10Z-heptadecenoic acid) and the overall upregulation of Cluster 1 (mean log_2_FC = 0.385), which was significantly enriched in the linoleic acid metabolism pathway. Second, during the critical transport and flux-maintenance phases, males exhibited enhanced mitochondrial uptake and degradation capacity. Acylcarnitines are essential carriers for transporting fatty acids into the mitochondria for β-oxidation, and elevated levels typically reflect increased oxidative flux [37]. The significant increases in long-chain acylcarnitines, such as oleoylcarnitine (C18:1) and palmitoylcarnitine (C16:0), indicate efficient transport via the “carnitine shuttle“ [38]. Simultaneously, acetylcarnitine (C2:0), which reflects the Acetyl-CoA pool, also rose significantly. The concurrent increase in long-chain acylcarnitines (reflecting transport) and short-chain acetylcarnitine (reflecting degradation products) provides direct evidence of fatty acid oxidation progressing from transport to degradation. This efficient oxidative flux serves as the molecular driver for the superior fat reduction observed in males. Furthermore, males exhibited systemic metabolic integration; the ketone body 3-hydroxybutyrate (No. 58) showed the most dramatic fold change in males, signaling an active process where the liver converts surplus oxidation products into ketones for peripheral release [39].

Concurrently, males exhibited a “protein-sparing” tendency. Previous studies have demonstrated that through the Randle cycle mechanism, when fatty acid oxidation provides sufficient energy substrates, it inhibits the oxidation and utilization of glucose [40]. Subsequent research has further confirmed that such substrate competition similarly impacts amino acid remodeling and metabolism [41]. Our results showed that Cluster 2 (rich in amino acid pathways) was downregulated in both sexes, but the magnitude was significantly greater in males (log_2_FC = −0.333). This more pronounced downregulation in males is likely a physiologically meaningful “protein preservation effect“ [42], whereby the body reduces the entry of amino acids into catabolic pathways when fatty fuel is abundant, thereby protecting functional tissues like muscle during intensive fat loss.

### Females favor prioritized regulation of amino acid metabolism networks during short-term training program

In contrast to the full-chain fatty acid oxidation observed in males, our results suggest that female obese adolescents prioritize the regulation of amino acid metabolism networks.

Females demonstrated relatively conservative lipid mobilization characteristics. PCA plots (Fig. 1A-C) and volcano plots indicated that females lack the numerous and dramatic lipid upregulation signals seen in males. Furthermore, in Cluster 1 (fatty acids and acylcarnitines), the response intensity in females (log_2_FC = 0.028) was substantially lower than in males (log_2_FC = 0.385). these findings are highly consistent with the clinical observation that fat reduction in females is less pronounced than in males [43], suggesting a stronger adipose preservation mechanism in females under exercise stress.

Simultaneously, females exhibited prioritized remodeling of amino acid networks. While both sexes showed a downregulation of amino acid catabolism in Cluster 2, the magnitude in females (log_2_FC = −0.077) was much more stable compared to the dramatic fluctuations in males (log_2_FC = −0.333). This steadier trend reflects a “thrift effect” on the endogenous protein and amino acid pool. Amidst this general stability, females specifically increased the levels of gluconeogenic amino acids, such as asparagine (No. 2), threonine, and serine (No. 15) (Fig. 2C). This strategy of “global thrift with local supply” provides a controlled “internal substrate” for gluconeogenesis. It suggests that females maintain glucose homeostasis by optimizing endogenous glucose precursors rather than relying on intensive fatty acid oxidation, thereby effectively reducing secretory pressure on pancreatic β-cells. This molecular profile aligns with the significant improvement in HOMA-β (*P =* 0.010) observed in females. Additionally, pathway enrichment indicated that female metabolic remodeling is highly focused on core amino acid networks. For instance, the rise in β-alanine suggests that female muscle tissue favors intracellular fatigue-resistance (e.g., carnosine synthesis) to adapt to stress, rather than sacrificing amino acids for explosive energy expenditure as seen in males.

### Predictive modeling suggests predictable improvements in females and predictive insufficiency in males

To quantify the link between changes in sex-differential metabolites and clinical improvements, we constructed sex-differential PLS models (Table 6).

The PLS models successfully predicted glucolipid improvements in females. The 65 sex-differential metabolites effectively predicted the improvement of LDL-C (Q^2^ = 0.1853) and FBG (Q^2^ = 0.1321) in females, suggesting a detectable association between the restoration of glucolipid homeostasis and the remodeling of circulating metabolites (particularly amino acids). Pathway enrichment revealed that the networks driving glucose improvement (Fig. 4C) and lipid improvement (Fig. 4D) share tryptophan metabolism but also exhibit functional difference (e.g., LDL-C improvements were linked to D-amino acid metabolism, while FBG improvements involved broader amino acid branches). However, the PLS model could not predict fat loss in females (Q^2^ < 0), consistent with the idea that the metabolic priority for females in this short-term intervention is amino acid regulation rather than adipose depletion.

Conversely, the PLS models showed insufficient predictive capacity for males. The model for male FBG showed only weak predictive power (Q^2^ = 0.0091), far lower than that in females. Despite the superior fat-loss results in males, the metabolites failed to predict changes in fat mass, body fat percentage, or weight (Q^2^ < 0). We interpret this predictive insufficiency as an indication that the dramatic fluctuations in male metabolites represent a more diffuse, systemic metabolic network characterized by high-flux energy turnover during intensive exercise. Because this response is molecularly widespread, its movements are not perfectly synchronized with single clinical indicators, leading to model failure. This further suggests that the remarkable fat loss and glucolipid improvements in males may stem from a more systemic lipid mobilization network that transcends the differential metabolites identified here.

### Future perspectives and limitations

This study reveals clear sexual dimorphism in the response of obese adolescents to short-term Training Program. The identified strategies—synergistic fatty acid oxidation and protein sparing in males versus prioritized amino acid network regulation in females—provide a new perspective on exercise adaptation. However, several limitations remain:

First, due to the modest sample size and intervention duration, these conclusions require validation in larger populations. This 4-week program primarily captures early-stage adaptation; longitudinal studies (e.g., 3–6 months) are needed to determine if the slower fat-loss efficiency in females improves or even surpasses that of males over time. Furthermore, as plasma samples were collected within 24 h of the final exercise bout, the results may include residual acute short-term Training Program effects rather than purely long-term adaptive remodeling.

Second, the training program in this study was strenuous and conducted under strict residential supervision. While achieving excellent short-term metabolic gains, the long-term compliance and sustainability of such a high-intensity regimen in daily life may be challenging for adolescents. Future research should explore more moderate, sex-differential exercise prescriptions.

Additionally, as this study relies on circulating metabolomics, it cannot definitively localize the tissue source (e.g., skeletal muscle, adipose, or liver) of the observed metabolic changes. Future studies utilizing invasive tissue biopsies or metabolic tracers are warranted. Finally, the relationships established here are correlational; animal models or in vitro functional experiments (e.g., intervening on β-cell function with gluconeogenic amino acids) are necessary to verify causality.

## Conclusion

Obese adolescents exhibit significant sexual dimorphism in their response to short-term Training Program. Under the same standardized training program, males achieve greater fat reduction and appear to leverage a synergistic strategy of fatty acid oxidation and protein sparing, whereas females prioritize amino-acid network fine-tuning. Predictive modeling indicates that sex-differential metabolites can predict glucolipid improvements in females. These findings provide a scientific basis for developing sex-differential exercise prescriptions for metabolic health.

## Supplementary Information

Supplementary Material 1.

Supplementary Material 2.

## Data Availability

The datasets used and/or analysed during the current study are available from corresponding authors on reasonable request.

## Declarations

### Related publications

The cohort and intervention protocol described in the present study have been previously reported in related publications focusing on different research objectives, including overall metabolomic changes following the intervention, insulin resistance phenotypes, proteomic remodeling, and inter-individual variability in weight loss response. The current manuscript addresses a distinct scientific question, specifically sex-differential metabolic responsiveness to short-term exercise intervention, employing linear mixed-effects models and predictive metabolomic modeling approaches that were not applied in the previous studies.There is no duplication of primary results, statistical models, or interpretations between the present manuscript and prior publications.
