# Supplementary material for: Sex differences in response to a short-term training program intervention in obese adolescents: a plasma metabolomics study
Source: Biol Sex Differ. 2026 Apr 1;17:103. doi: 10.1186/s13293-026-00896-8 (PMC13169799; doi:10.1186/s13293-026-00896-8)
Supplement: Supplementary file 1 — Supplementary Material 1. [file 13293_2026_896_MOESM1_ESM.pdf]

Informed Consent Form-Informed Consent Page

Protocol Name: Scientific Research on Exercise for Fat Reduction in Obese Adolescents

Research Institution: Guangzhou Sport University

Principal Investigator: Lin Zhu

Address: Academic of Sport and Health, Guangzhou Sport University, No. 1268 Guangzhou Avenue Middle, Tianhe District, Guangzhou City, Guangdong Province, China

Dear Participant,

You are being invited to participate in a scientific research study on exercise for fat reduction in obese adolescents. Before you decide whether to participate in this study, please read the following content carefully. It will help you understand the study, its purpose, procedures, and duration. If you wish, you may discuss it with your family members or friends, or ask the research staff for explanations to assist you in making your decision.

I. Research Background and Objectives

Obesity poses a serious threat to the physical health of adolescents. Numerous studies suggest that obesity is often accompanied by metabolic disorders, leading to a trend of younger onset of metabolic diseases such as diabetes. Changes in human metabolism are usually accompanied by alterations in endogenous small molecule metabolites. Identifying metabolic biomarkers of metabolic disorders in obese adolescents has a positive role in developing targeted intervention programs. The obese adolescent population has its uniqueness; their energy expenditure and intensity during various physical activities may differ significantly from those of normal-weight adolescents. Understanding the energy expenditure and intensity of various physical activities in obese adolescents is crucial for formulating exercise intervention programs. Exercise increases energy expenditure, thereby reducing fat accumulation in obese adolescents and promoting metabolic health. However, the quantitative relationship between exercise and fat reduction in obese adolescents, as well as whether exercise promotes metabolic health in obese adolescents by altering endogenous small molecule metabolites, remains unclear.

Therefore, this study aims to identify biomarkers of metabolic abnormalities in obese adolescents by assessing changes in endogenous small molecule metabolites in normal-weight, obese, and obese adolescents with metabolic abnormalities. Objective measurements will be conducted on the energy metabolism characteristics of obese adolescents during various physical activities to clarify the energy expenditure and intensity of different physical activities. Based on this, exercise interventions will be implemented to determine the relationship between exercise, fat reduction, and metabolic health in obese adolescents, further clarifying the biomarkers through which exercise promotes metabolic health in obese adolescents. This will provide a

theoretical reference for developing scientific and effective strategies to prevent and treat metabolic abnormalities in obese adolescents.

II. What You Will Need to Do if You Participate in the Study

Before enrolling in the study, you will undergo the following examinations to determine your eligibility. The research staff will conduct tests on your body morphology, body composition, energy expenditure under resting and exercise conditions, pubertal assessment, and medical examinations. You need to follow the instructions of the research staff to complete these tests. All test items are non-invasive, and you may terminate the tests at any time if you feel uncomfortable.

Based on your test results, the research staff will develop a personalized fat reduction program for you and implement scientific and effective weight loss interventions. You only need to cooperate with the research staff to complete your intervention program and related tests.

After the completion of the tests, your personal information will be stored intactly at the research institution. Any public reports related to the study results will not disclose your personal information. Every effort will be made to protect your personal data. Participation in the study is entirely voluntary. You may refuse to participate in this study or withdraw from it at any time during the study without facing discrimination or retaliation, and your rights and benefits during the study period will not be affected.

Informed Consent Form-Consent Signature Page

Research Project Name: Scientific Research on Exercise for Fat Reduction in Obese Adolescents

Sponsor: Lin Zhu

Consent Statement

I have read the above introduction to this study and have had the opportunity to discuss it with the research staff and ask questions. All my questions have been satisfactorily answered.

I understand the potential risks and benefits associated with participating in this study. I acknowledge that participation is voluntary, and I confirm that I have had sufficient time to consider this. I understand that:

I can ask the research staff for more information at any time.

I can withdraw from this study at any time without facing discrimination or retaliation, and my rights and benefits during the study period will not be affected.

I consent to the ethics committee or the sponsor's representative accessing my research data.

I will receive a signed and dated copy of this informed consent form.

Finally, I have decided to consent to participate in this study and will try my best to follow the medical advice.

Participant's Signature (Handwritten): _______________

Date: _______________

Participant's Contact Number: _______________

Participant's Legal Representative's Signature (if necessary) (Handwritten): ________

Date: _______________

Representative's Contact Number: _______________

Investigator's Statement

I confirm that I have explained the details of this study, including the participant's rights, potential benefits, and risks, to the patient and have provided them with a signed copy of the informed consent form.

Investigator's Signature (Handwritten) (Informing the Participant): _______________

Date: _______________

Investigator's Contact Number: _______________
