# Supplementary material for: Sex differences in response to a short-term training program intervention in obese adolescents: a plasma metabolomics study
Source: Biol Sex Differ. 2026 Apr 1;17:103. doi: 10.1186/s13293-026-00896-8 (PMC13169799; doi:10.1186/s13293-026-00896-8)
Supplement: Supplementary file 2 — Supplementary Material 2. [file 13293_2026_896_MOESM2_ESM.pdf]

广州体育学院人体实验伦理审查表

Human Experimental Ethics Inspection of

Guangzhou Sport University

审批编号（ID Number）：2018LCLL-008

<table>
<tr><td>项目名称
Project name</td><td>肥胖青少年运动减脂科学化研究</td><td>项目来源
Source of project</td><td>国家社科基金</td></tr>
<tr><td>项目负责人
Project director</td><td>朱琳</td><td>实验方案
Experimental scheme</td><td>（请附件说明）</td></tr>
<tr><td>研究对象
Objects</td><td colspan="3">（年龄、性别、健康状况、人数、职业等。）
年龄：10-17 岁肥胖青少年。
性别：男女不限。
健康状况及人数：研究对象 BMI 符合《学龄儿童青少年超重与肥胖筛查》标准；单纯性肥胖，无身体、智力发育异常；无肥胖相关并发症，如心血管疾病等；无运动受限的相关疾病。
人数：120 人。
职业：中小学学生。</td></tr>
<tr><td>实验要点
Outline of experiments</td><td colspan="3">（包括实验周期、干预方法、观测手段和指标、涉及人体的取样或部位等。）
研究包含两部分：
第一部分为横断面研究：主要比较代谢综合征肥胖青少年与代谢正常肥胖青少年、正常体重青少年机体内源性小分子代谢物的差异，从而确定肥胖青少年代谢异常的生物标记物。
第二部分为干预类研究：通过对肥胖青少年进行 4-6 周的封闭减肥营干预，干预方法为运动结合饮食控制。
研究所涉及的观察手段和指标如下：
身体形态及身体成分测试：采用身高体重计测试受试者的身高和体重；采用围度尺测试受试者的身体围度；采用 T-scan 生物电阻抗测试受试者身体成分。
青春分期测试：采用《自填式青春发育量表》评估受试者的青春发育情况。
血压测试：采用欧姆龙电子血压计测试受试者的收缩压和舒张压。
血液生化检测：空腹血糖、空腹胰岛素、血脂四项（总胆固醇、甘油三酯、高密度脂蛋白胆固醇、低密度脂蛋白胆固醇）。
内源性小分子代谢物检测：采用质谱串联液相色谱检测血浆中内源性小分子代谢物的含量。
能量消耗测试：采用间接测热法和运动加速度计法测试肥胖青少年的能量消耗。</td></tr>
</table>

	**人体取样**：血浆和血清，取静脉血 5ml。
审查内容<br>Contents of inspection	1．研究方案、课题标书及其他支持性文件<br>2．知情同意书<br>3．招募研究对象的材料或广告<br>4．对研究对象因参加研究而给与的任何补偿、保险等说明<br>5．需由研究对象填写的表格和问卷<br>6．研究人员履历及联系方式
申请者声明<br>Announcement of applicant	我将自觉遵守人体实验的伦理原则，随时接受伦理委员会的监督与检查，如违反规定，自愿接受处罚。I will abide by the rules of human experimental ethics, accept the supervision and inspection of ethics committee, and accept the punishment if any infringement.<br>声明人签名 (Signature): [signature]<br>2018 年 6 月 25 日
审查结果<br>Results of inspection	广州体育学院伦理委员会审查意见：<br>☑同意 （Agree）<br>☐修改后同意（Agree after revised）<br>☐修改后重审（Retrial after revised）<br>☐不同意 （Disagree）<br>☐暂停或终止实验（Suspension or termination）<br>主任委员（签名）：[signature]<br>伦理委员会（盖章）[stamp: 广州体育学院伦理审查委员会]<br>2018 年 6 月 25 日

说明：

1. 审批编号须审查通过后，由伦理委员会填写；
2. 申请人若为在校研究生，导师为项目负责人；
3. 审查材料一式两份，相关审查资料随本表一并递交。
